# Blue-light photodegradation of ferricyanide under protein relevant conditions†

**DOI:** 10.1039/d4dt02916j

**Published:** 2025-02-07

**Authors:** Patrick D. F. Murton, Christiane R. Timmel, Stuart R. Mackenzie, Patricia Rodríguez-Maciá

**Affiliations:** a Department of Chemistry, University of Oxford, Chemistry Research Laboratory Mansfield Road Oxford OX1 3TA UK prm28@leicester.ac.uk; b Department of Chemistry, University of Oxford, Inorganic Chemistry Laboratory South Parks Road Oxford OX1 3QR UK; c School of Chemistry and Leicester Institute for Structural and Chemical Biology, University of Leicester University Road Leicester LE1 7RH UK

## Abstract

Ferricyanide is commonly used as a reoxidant in photochemical studies of redox proteins including cytochromes, photosystem II and flavoproteins. A low-spin *d*^5^ complex, [Fe(iii)(CN)_6_]^3−^ is a powerful electron acceptor which efficiently reoxidises photo-generated radical species. Unfortunately, ferricyanide itself absorbs strongly in the blue and a better understanding of its own photochemistry is required. Here, we present a combined UV/Vis and infrared spectroscopic study of the blue light photo-induced degradation of ferricyanide under conditions commonly employed in photochemical studies of proteins. Clear differences are observed in the photochemistry in pure water, Tris buffer and 20% glycerol solution, which are interpreted in terms of solvent–ligand exchange and ligand to metal charge transfer. The implications for photochemical studies of proteins employing ferricyanide as a reoxidant are discussed.

## Abstract 2

Blue light photo-induced degradation of ferricyanide under protein relevant conditions shows different solvent–ligand exchange and ligand to metal charge transfer. The presence of glycerol appears to result in more photostable products.

## Graphical abstract

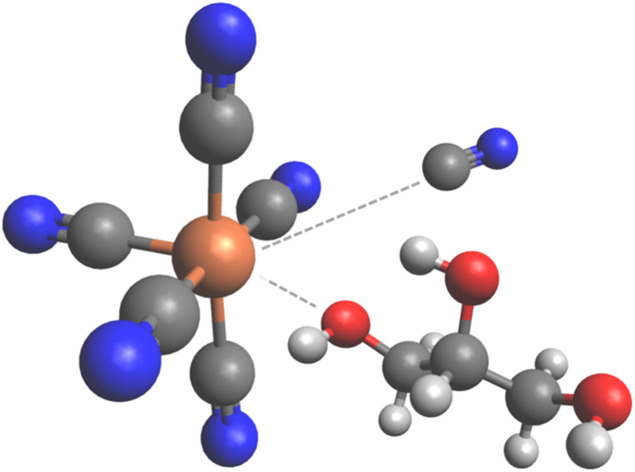

## 1. Introduction

In the study of redox proteins it is common practice to add oxidising and/or reducing agents to initiate redox processes, to act as tractable redox partners or to aid in sample recovery following long-lived product formation.^1–12^ One commonly used oxidising agent is potassium ferricyanide, K_3_[Fe(iii)(CN)_6_], which is often employed in the study of proteins such as complex 1, laccase, hydrogenase, cytochrome, photosystem II and flavoproteins.^1,13–22^ Of particular note, potassium ferricyanide is widely used in photochemical studies of cryptochromes – blue-light receptor flavoproteins which are believed to play a central role in the magnetic sense of animals, especially night migratory songbirds.^18,23^ In such studies, the role of ferricyanide is to accelerate the reoxidation of long-lived neutral radicals that are formed in the photocycle.^18,21,24–28^

Ferricyanide is a paramagnetic low-spin *d*^5^ octahedral (O_h_) complex used in a variety of applications from redox flow batteries to glucose sensing.‡‡In some literature^29^ the unevenly populated t_2g_ orbitals are thought to induce Jahn–Teller distortion leading to *D*_4h_ or *D*_3d_ ^30^ symmetry. Nonetheless, an octahedral symmetry is assumed in the following analysis.^31–34^ Importantly, it readily acts as an electron acceptor, forming the low-spin diamagnetic ferrocyanide complex, [Fe(ii)(CN)_6_]^4−^.^35,36^

Although widely used in photochemical studies of proteins, ferricyanide is known to undergo its own ultrafast photochemistry in water following absorption at *λ* < 430 nm. For example, excitation of the first two absorption bands (see Fig. S2†) centred at *λ* ≈ 420 nm (^2^T_2g_ → ^2^T_1u_ band, *ε* ≈ 1040 M^−1^ cm^−1^), respectively, induces fast ligand-to-metal charge transfer (LMCT) followed by back electron transfer within 0.5 ps in water.^29,31,36,37^ This rapid intramolecular deactivation is highly efficient in ferricyanide (much more so than in ferrocyanide) with almost all excited molecules returning rapidly to the ground state. The only other reaction channel, with a quantum yield *Φ* < 0.02, involves CN^−^ loss followed by aquation.^36–41^ The ultrafast mechanism for this ligand exchange process is still the subject of study and depends on the photoexcitation wavelength and thus which excited states are accessed.^36,38,42,43^ Reinhard *et al*. recently assigned a ^2^[Fe(iii)(CN)_5_]^2−^ intermediate and ^2^[Fe(iii)(CN)_5_OH_2_]^2−^ product following 336 nm excitation to the ^2^T_2u_ excited state.^38^ Ojeda *et al*., by contrast, observed [Fe(ii)(CN)_5_OH_2_]^3−^ as a direct product of 400 nm photo-induced LMCT in ferricyanide. Both potential degradation products were predicted decades ago^42–44^ and although some aspects of the ultrafast dynamics remain uncertain^35,41^ there is general agreement that photoaquation represents a minor, but important, side reaction in the near-UV ferricyanide photocycle.

Given the role of ligand exchange in ferricyanide photochemistry, it is unsurprising solvent conditions play a key role. Markedly different chemistry is observed in solvents other than water where different potential ligands exist and the efficiency of geminate recombination is affected.^37,45^ To our knowledge, however, ferricyanide photochemistry has not been studied under conditions typically used in protein photochemistry nor has ferricyanide photochemistry figured prominently in the protein photochemistry literature.^18,20,28^

In attempts to measure small magnetic field effects in cryptochromes, in Oxford we have recently developed a range of sensitive direct absorption based techniques employing high finesse optical cavities.^18,25,26,46–49^ With one such technique, broadband cavity-enhanced absorption spectroscopy (BBCEAS), potassium ferricyanide photochemistry complicates the photoinduced absorption spectra of cryptochrome proteins under continuous photoexcitation at 450 nm, (see Fig. S1 and S3†). Specifically, a broad absorption band (500–700 nm) is observed to grow in over an illumination period of 10 s. Although ferricyanide absorbs weakly at 450 nm (ESI Fig. S2,†*ε* ≈ 250 M^−1^ cm^−1^), even low yields of a stable degradation product can accumulate over long time scales (ms–min) and compete with detection of the protein photochemistry of interest, especially in highly sensitive experiments such as BBCEAS.

In this study we explore the photodegradation process(es) of potassium ferricyanide under conditions typically employed in the study of flavoproteins and other proteins (*e.g.*, pH 8 Tris buffer and 450 nm excitation). A series of UV/Vis and infrared (IR) absorption experiments were carried out under continuous irradiation in order to monitor the formation of degradation products (UV/Vis) and their oxidation states (IR). To help interpret the experimental data, density functional theory (DFT) calculations have been performed to assess the stability of potential degradation products and plausible mechanisms for their formation. One major aim of this paper is to highlight the potential perils of ferricyanide and its photodegradation products when the former is used in the study of proteins and protein photochemistry.

## 2. Methods

### 2.1. UV/Vis and IR experiments under continuous irradiation

All irradiation experiments involved continuous illumination of a sample with the output of a light emitting diode (LED) over a long time period (s–h) combined with spectroscopic analysis (ESI Fig. S4†). In this instance, a 450 (±18) nm LED was used to simulate the excitation wavelength employed when studying flavoproteins. The system was then probed in the visible and IR spectral regions. In both cases the samples were illuminated with 20 mW of light. However, due to the different spatial restrictions in the two spectrometers, the exact irradiance differed between them with the UV/Vis spectrometer allowing for more homogeneous illumination.

Solution-based UV/Vis absorption experiments were performed on 125 μL samples of 1 mM ferricyanide (Sigma Aldrich) in different buffers and solvents common to protein photochemical studies (*e.g.*, use of Tris buffer and/or glycerol, see Results section for details). Ferricyanide samples were placed in a 1 cm pathlength quartz sample cell (Hellma Z600334) inside an Agilent Cary 60 spectrometer with spectra recorded, at room temperature, from 200 nm–750 nm with 1 nm resolution. Spectra were recorded using the internal Cary WinUV software (Agilent) with 1 min intervals for the first hour and 30 min intervals thereafter. All acquired data was subsequently processed in Matlab.

Transmission IR experiments were performed on 5–6 μL samples of 5 mM ferricyanide (Sigma Aldrich) in the same buffers/solvent combinations (see Results section). The aqueous ferricyanide samples were sandwiched between two CaF_2_ windows (31.8 mm diameter, 1.5 mm thickness, Crystan) separated by a 50 μm Teflon spacer (Kromatek Ltd) in a commercial IR transmission cell (PIKE). Spectra were measured at room temperature inside an anaerobic, dry glovebox (Glove Box Technology Ltd, <2 ppm of O_2_, <85 °C dew point) on a Bruker Vertex 80 FTIR spectrometer equipped with a mercury cadmium telluride (MCT) detector cooled with liquid N_2_. Each spectrum was recorded as an average of 1024 interferograms using OPUS software (Bruker) in the double-sided, forward–backward mode with a resolution of 2 cm^−1^, an aperture setting of 1 mm and a scan rate of 20 Hz. The IR data were baseline corrected by spline interpolation, excluding the peaks, and subsequent subtraction from the raw data.

### 2.2. Density functional theory

Density Functional Theory (DFT) calculations were performed following a procedure similar to that employed by Ross *et al*.^50^ The PBE0 hybrid exchange–correlation functional was employed in conjunction with the 6-311G** basis set for all non-metal atoms. Meanwhile, the Stuttgart/Dresden (SDD) electron core potential was combined with the def2-TZVP basis set to describe the Fe atom. The solvent was accounted for by a simple polarisation continuum model (SMD) with water used in each case. All calculations were run in ORCA 5.0 with results visualised in Chemcraft 1.8.

## 3. Results

### 3.1. Potassium ferricyanide photochemistry in pure water

**Fig. 1:**
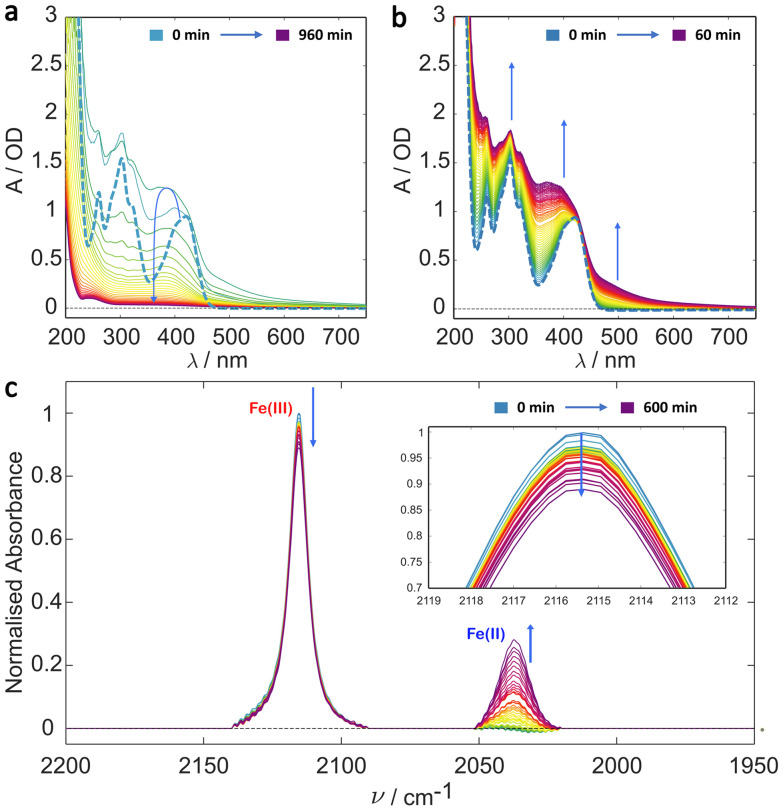
Irradiation studies of potassium ferricyanide in deionised water. (a) Evolution of the UV/Vis spectrum of 1 mM ferricyanide solution during irradiation over a 16 h period with spectra shown at 30 min intervals. The kinetics appears to be A → B → C. (b) UV/Vis spectra over the first hour of illumination with spectra recorded every minute. In both (a) and (b) the bold dashed line is the *t* = 0 ferricyanide spectrum. (c) Evolution of the IR spectrum of 5 mM ferricyanide solution during continuous irradiation over a 10 h period with a spectrum taken every 15 min, indicating some Fe(ii) formation (2037 cm^−1^) from Fe(iii) (2115 cm^−1^). The inset in (c) highlights the decay of the top of the Fe(iii) CN stretch. Arrows indicate the signal change with time (*i.e.* growth or decay).

Building upon previous continuous illumination studies and recent ultra-fast spectroscopy studies,^29,36–38,42^ initial irradiation experiments were performed on solutions of potassium ferricyanide in deionised water. The UV/Vis results are shown in Fig. 1a and b. The dominant ^2^T_2g_ → ^2^T_1u_ and ^2^T_2g_ → ^2^T_2u_ bands are clearly visible centred at 420 nm and 300 nm, respectively in the *t* = 0 spectrum (see also Fig. S2†). Over the total 16 h illumination period the system exhibits complex A → B → C type kinetics (Fig. 1a and Fig. S10a†). During the first hour of illumination (Fig. 1b), there is a clear growth in the absorbance below 600 nm and particularly between the spectral bands of ferricyanide (200–400 nm and >450 nm) consistent with the initial formation of the aquapentacyanoferrate species. With continued illumination, however, this species, along with the ferricyanide itself decay further leaving a clear solution with negligible absorbance in the visible.

Photoinduced reduction of the metal centre is confirmed by the IR detected irradiation experiment, the results of which are shown in Fig. 1c. This shows a clear decrease in the parent Fe(iii) band at 2115 cm^−1^ and simultaneous production of a new Fe(ii) band at 2037 cm^−1^. This assignment is unambiguous. The triply degenerate ^1^T_1u_ CN stretch occurs in ferricyanide and ferrocyanide complexes at 2115 cm^−1^ (*ε* ≈ 784 M^−1^ cm^−1^)^50^ and 2037 cm^−1^ (*ε* ≈ 2373 M^−1^ cm^−1^) respectively making them easily distinguishable. The red-shift in the ferrocyanide complex compared with the ferricyanide, arises from the stronger π* back donation from the more electron rich Fe(ii) metal centre, weakening the CN bond.^31^ The growth (from zero) of the peak centred at 2037 cm^−1^ indicates the formation of Fe(ii) in support of an [Fe(ii)(CN)_5_OH_2_]^3−^ photoproduct.^36^ However, the CN stretch in Fe(ii) complexes has a much higher extinction coefficient than that in ferricyanide (*ε*_Fe(II)_ ≈ 2373 M^−1^ cm^−1^*vs*. *ε*_Fe(III)_ ≈ 784 M^−1^ cm^−1^ in the respective hexacyano complexes) and so the limited growth in the 2037 cm^−1^ band indicates little of this oxidation state accumulates, even over these extended timescales.

**Scheme 1:**
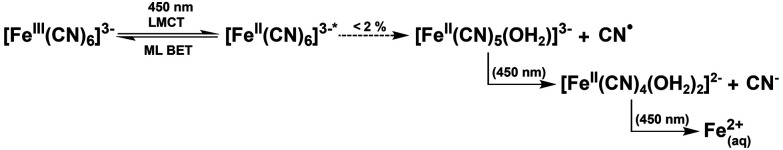
Photochemical degradation of ferricyanide in water. * indicates electronic excited state.

Importantly, the observed changes in both the UV/Vis and the IR spectra occur only under blue light illumination with neither spectrum showing any measurable change over a period of 10 h otherwise (ESI Fig. S5 and S6,† special care was taken to measure the control experiments under dark conditions). The evolution of both spectra is consistent with A → B → C kinetics summarised in Scheme 1 in which we identify B as the [Fe(ii)(CN)_5_OH_2_]^3−^ complex, most likely formed by cyano radical loss from the LMCT excited state of ferricyanide.^45^ The same product is formed much more efficiently by photoinduced CN^−^ loss from ferrocyanide ([Fe(ii)(CN)_6_]^4−^).

These results are in line with observations made by Moggi *et al*.,^42^ and others,^36,43,51–56^ who further observed that the pH of the ferricyanide solution in pure water increased during illumination. This is likely due to the release of CN^−^ and CN˙ following photolysis. Indeed, it has been observed that, under illumination, the pH increased gradually in conjunction with an increase in the amount of free cyanide and decrease in the concentration of ferricyanide.^42,51,52,54^ Furthermore, the photolysis rate or yield was generally seen to increase with increasing pH, potentially arising from an increase in the CN^−^ ligands electron density promoting LMCT and a shift in the Fe(iii)/Fe(ii) redox couple.^36,42,43,51,54,57^

Another plausible degradation mechanism involves the formation of water-insoluble Prussian blue (Fe(iii)_4_[Fe(ii)(CN)_6_]_3_·*x*H_2_O), which is known to occur in aqueous mixtures of ferricyanide and ferrocyanide.^42^ Given that photoaquated ferrous cyano complexes appear to be formed, it follows that Prussian blue will eventually be produced leading to a colourless solution consistent with the results shown in Fig. 1. This reaction has been observed in continuous white-light irradiation experiments and is a known problem when doing photochemistry with ferricyanide since the insoluble Prussian blue causes laser scattering during measurements.^37,42^ In addition, it is possible that the continued degradation may lead to the formation of mixed-hydroxo species, especially since the pH will increase during illumination.^42^ This would also likely lead to an insoluble precipitate and thus colourless solution.

It is clear then that, in pure aqueous solution, significant and complex ferricyanide photochemistry occurs over these prolonged time scales, even when illuminated at 450 nm, in the extreme tail of the lowest energy absorption band. These results provide a baseline for studies under conditions typically employed for protein photochemical studies.

### 3.2. Potassium ferricyanide photochemistry in Tris buffer with 20% glycerol (v/v)

**Fig. 2:**
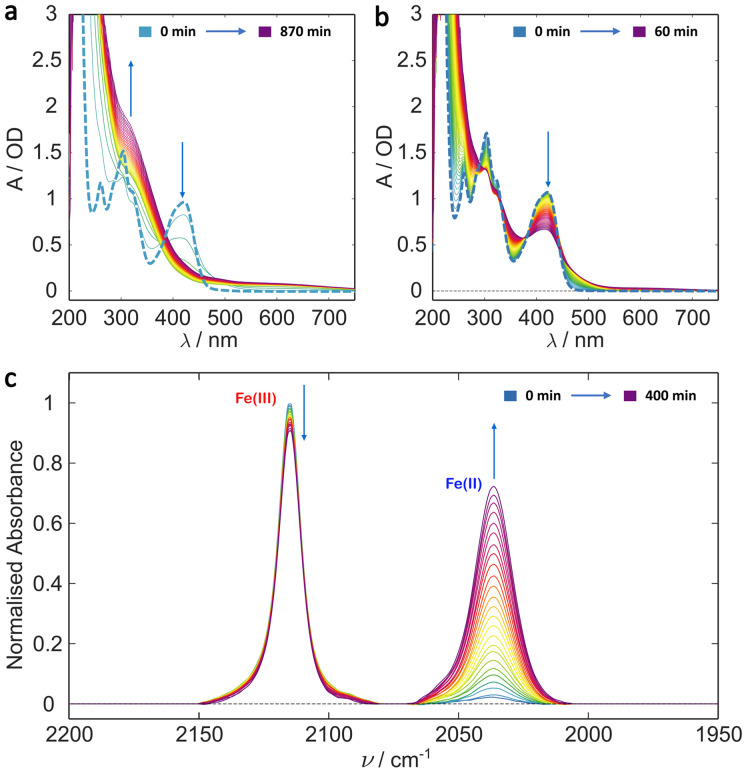
Irradiation studies of potassium ferricyanide in Tris buffer with 20% glycerol (v/v). (a) UV/Vis spectra of 1 mM ferricyanide solution recorded every 30 min over a 14.5 h period. (b) UV/Vis irradiation over the first hour with a spectrum taken every minute showing clear isosbestic points indicating simple A → B kinetics for at least the first hour. In both (a) and (b) the bold dashed line is the *t* = 0 ferricyanide spectrum. (c) Evolution of the IR spectrum of 5 mM ferricyanide solution over a 7 h irradiation period with spectra taken every 15 min, indicating clear reduction of the metal centre. Arrows indicate direction of signal change with time (*i.e.* growth or decay).

Many protein photochemical experiments are performed under more physiologically relevant solution conditions. In the case of cryptochromes these often involve pH 8 solution achieved using Tris (tris(hydroxymethyl)aminomethane) buffered saline solution.^18,58–60^Fig. 2 shows the results of irradiating ferricyanide in pH 8 Tris buffer (10 mM Tris, 250 mM NaCl) with 20% glycerol (v/v). The photochemistry is markedly different to that in pure water indicating different kinetics and/or photoproducts. The UV/Vis irradiation data (Fig. 2a and b) show the initial formation of some photoproduct with apparently simple A → B kinetics, particularly in the first hour, as indicated by the clear isosbestic points (*e.g.*, at 380 nm and 450 nm). In this case, unlike in water, the absorbance below 400 nm continues increasing with illumination beyond the first hour and a weak persistent tail to the absorbance spectrum grows in out to 700 nm (Fig. 2a). This suggests that the photobleaching process observed in water is arrested in the Tris/glycerol solution. The product spectrum, at least below 400 nm, is similar to that of ferrocyanide (Fig. S2†) but the relative extinction coefficients of ferricyanide (*ε*_420 nm_ ≈ 1040 M^−1^ cm^−1^) and ferrocyanide (*ε*_320 nm_ ≈ 318 M^−1^ cm^−1^), suggest that the product signal is too large to be purely the latter. Nor can the production of ferrocyanide account for the long wavelength tail which presents a particular problem for cryptochrome studies as it obscures the spectral region in which photogenerated radicals such as the neutral protonated flavin adenine dinucleotide radical (FADH˙) and tryptophan radicals absorb.^61^

As in the deionised water study, the growth of an IR band centred at 2037 cm^−1^ indicates the photoreduction of the metal centre from Fe^3+^ to Fe^2+^ (Fig. 2c). However, in Tris buffer, the Fe(ii) peak is significantly larger than observed in the deionised water experiments (*cf.*Fig. 1c, peak at 2037 cm^−1^). Coupled with the fact that no evidence for secondary reactions is observed (Fig. 2a and b), this implies comparative photostability of the Fe(ii) product generated under these conditions.

Control experiments again showed minimal change in the UV/Vis or IR spectral signatures over a period of 10 h (ESI Fig. S7 and S8†) in the absence of photoexcitation, confirming the process observed in Fig. 2 is photoinduced.

Evidently, the photochemistry of ferricyanide differs significantly in 20% glycerol (v/v) Tris buffer *versus* deionised water. Both result in initial photoreduction of the metal centre by LMCT, followed by CN loss. However, where sequential aquation is believed to occur in pure water, an alternative, more photostable product appears to form in this case. Given the buffer composition, there are two candidate ligands, glycerol and Tris, both of which could bind *via* hydroxy groups (or a primary amine group in the case of Tris, ESI Fig. S9†). The relative binding energy of these complexes is considered below.

### 3.3. Potassium ferricyanide photochemistry in 20% glycerol (v/v)

**Fig. 3:**
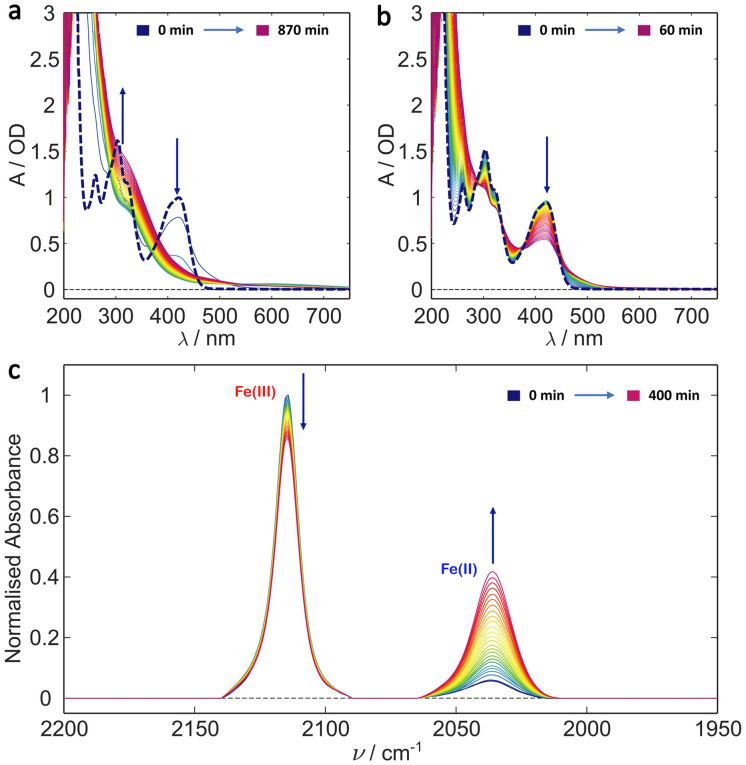
Potassium ferricyanide irradiation in 20% glycerol/water (v/v). (a) UV/Vis spectra of 1 mM ferricyanide solution shown at 30 min intervals during a 14.5 h illumination period. (b) UV/Vis irradiation over the first hour with a spectra shown every minute. The kinetics appears to follow simple A → B kinetics for at least the first hour. In both (a) and (b) the bold dashed line is the *t* = 0 ferricyanide spectrum. (c) Evolution of the IR spectrum of 5 mM ferricyanide solution over a 7 h period with spectra taken every 15 min. Clear formation of Fe(ii) from Fe(iii) is observed. Arrows indicate direction of signal change with time (*i.e.* growth or decay).

**Scheme 2:**
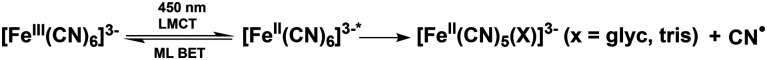
Photochemical degradation of ferricyanide in glycerol solution where the asterisk indicates an electronic excited state. Subsequent photodegradation is arrested compared to pure water.

In order to determine the individual effects of the Tris buffer and the glycerol the photoillumination experiments were repeated on ferricyanide in 20% glycerol and deionised water (v/v). The results are shown in Fig. 3. Both the UV/Vis and IR results are similar to those recorded in the Tris buffer, with glycerol, above (*cf.*Fig. 2). That is, irradiation induces photoreduction forming an apparently photostable product which persists and whose concentration accumulates in the UV/Vis spectra with strong absorbance in the blue/near UV and weaker absorbance out to 700 nm. It seems likely that the same photoproduct forms here, even in the absence of Tris, suggesting either the binding of a glycerol molecule ([Fe(ii)(CN)_5_(glyc)]^3−^) or that the higher viscosity solution leads to more efficient geminate recombination, reducing photodegradation in general (see Scheme 2). The reduced absorption of the 2037 cm^−1^ CN stretch indicates lower Fe(ii) product yield *versus* the sample in Tris buffer and could arise from the change in pH and/or the reduced ionic strength (*e.g.* in the absence of Tris and sodium chloride). As mentioned, the formation of [Fe(ii)(CN)_5_OH_2_]^3−^ in aqueous conditions has been previously shown to be pH dependent with its yield likely promoted in more alkaline solutions.^36,42,43,51,54^ Therefore, it is possible that the yield of a glycerol-bound photoproduct is promoted in the more alkaline Tris buffer solution (pH 8) compared to simple 20% glycerol solution (initial pH 6.5).

### 3.4. Density functional theory

**Fig. 4:**
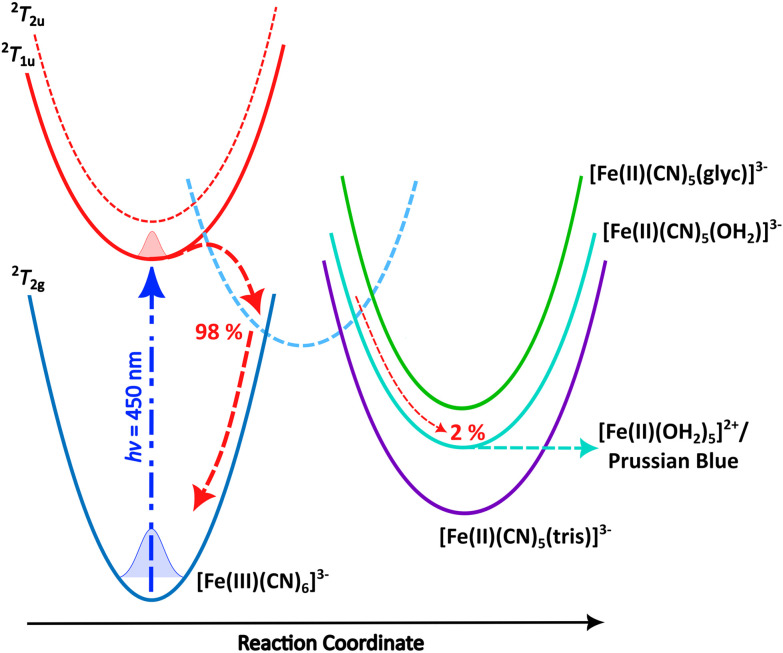
Schematic mechanism for the 450 nm photochemistry of ferricyanide. Initial excitation drives LMCT and rapid back electron transfer. Most complexes make it back to the electronic ground state within *ca.* 1 ps, but a small fraction undergoes ligand exchange. In pure water, further photoaquation of the resulting [Fe(ii)(CN)_5_(OH_2_)]^3−^ continues but this further photobleaching is arrested in 20% glycerol. Adapted from Reinhard *et al*.^38^

Photoreduction of ferricyanide clearly occurs in the various solvent systems above. By contrast no Fe^2+^ complexes were produced during photoillumination of ferricyanide in DMSO (Fig. S12†) highlighting the role of aquation in the mechanism. However, the process is clearly different – or at least has very different kinetics – in the presence of glycerol *versus* pure water. This could result from preferential binding of glycerol over water, leading to a more photostable Fe(ii) glycerol-bound photoproduct. To investigate this possibility, the binding energy and IR spectra of various potential photoproducts were calculated at the DFT level. Their relative energies are shown in Fig. 4 and listed in Table S2† while their simulated IR spectra (Fig. S11†) confirm that any of these products could give rise to the Fe(ii) CN stretch observed at 2037 cm^−1^ (see Fig. 1–3).

Both Tris and glycerol can bind to the Fe(ii) centre with the former binding more strongly. However, at 20% (v/v), glycerol is much the more likely to take the vacant site following CN˙ radical loss from the LMCT state in ferricyanide. The binding is sufficiently strong that, once bound it is unlikely to be displaced by water in its ground state and the negligible CN^−^ concentration in solution precludes the reverse exchange. Less clear is the effect complexation with glycerol might have on subsequent photodegradation. It is clear from the results in Fig. 1 and the literature^42,45^ that [Fe(ii)(CN)_5_OH_2_]^3−^ undergoes efficient further aquation following CN^−^ loss from its own excited state. However, the evidence from Fig. 2 and 3 suggests either that the [Fe(ii)(CN)_5_(glyc)]^3−^ product is more photostable or that the presence of glycerol reduces the quantum yield for all photodegradation processes.

## 4. Discussion

It is clear that the solvent conditions have a material effect on the photochemistry of ferricyanide, a better understanding of which is required to ensure these do not mask other photophysical processes where ferricyanide is employed as a reoxidant. The photodegradation of ferricyanide in water has received considerable attention and is reasonably well understood. Initial photoaquation following CN˙ radical loss generates the reduced [Fe(ii)(CN)_5_OH_2_]^3−^ complex which undergoes further photoaquation *via* efficient loss of CN^−^.^39–41^ This behaviour is highlighted in the kinetic evolution of the UV/Vis spectra at 420 nm shown in Fig. S10a† where there is initial growth on a time scale of 20–30 min before subsequent decay over a timescale of *ca.* 245 min (see Table S1†). As described by Vaz Da Cruz *et al*.,^40^ following [Fe(ii)(CN)_5_(OH_2_)]^3−^ formation, the covalently bound water molecule acts as a σ donor, primarily mixing with the iron d_*z*^2^_ orbital, lowering its energy. This additional orbital interaction together with a reduction of the molecular symmetry splits the d_*z*^2^_ energy level due to contributions from H_2_O and CN σ donation and π* back-donation. Meanwhile, the d_*x*^2^−*y*^2^_ orbital and formally t_2g_ orbitals are largely unaffected, mixing minimally with the water orbitals. This results in more pronounced absorption >500 nm from a metal-centred (MC) transition into an anti-bonding orbital which still absorbs reasonably strongly under 450 nm illumination, thus promoting further ligand dissociation.^40^ Given the [Fe(ii)(CN)_5_(OH_2_)]^3−^ ground state absorption is known with the MC transition peaking close to 450 nm,^39,40,44^ the above argument agrees well with both the UV/Vis and IR irradiation data for this system. Much less well understood is the relative photostability and further degradation of the various aquated products – including the mechanism for Prussian blue formation – which leads ultimately to complete bleaching in the visible part of the spectrum (and hence colourless solutions, see Scheme 1).

Of particular interest here, the photodegradation of ferricyanide in the presence of glycerol is rather different to that in pure water and appears to stop after reduction of the metal centre and formation of a more photostable product – potentially a glycerol-bound complex, [Fe(ii)(CN)_*x*_(OH_2_)_*y*_(glyc)_*z*_]^(2−*x*)^. This photoproduct has reduced absorbance at 450 nm compared with ferricyanide but absorbs strongly below 400 nm and exhibits weak but persistent absorbance out to 700 nm (see Fig. 3a and b). The reduced 450 nm absorbance leads to sequentially less absorption/greater photostability over time, thereby arresting the photobleaching. It is possible that photoinduced chelation of the singly substituted [Fe(ii)(CN)_5_(glyc)]^3−^ occurs as has been shown in a variety of pentacyanoferrates.^62–64^ However, these studies typically utilised near UV irradiation (365 nm) and observed that the quantum yield for secondary ligand dissociation and chelation decreased significantly at higher wavelengths. Thus, while this may still be a minor process under our conditions, it is likely significantly suppressed due to the weak absorption at 450 nm. This is further supported by the A–B nature of the spectral evolution (see Fig. 3a and b), which suggests that the process stops following [Fe(ii)(CN)_5_(glyc)]^3−^ formation.

This simple kinetic evolution was observed for both samples in Tris buffer and 20% glycerol (v/v) as exemplified by their kinetic evolution at 420 nm (Fig. S10b and c†). In Tris buffer, the ferricyanide absorption band decays on a time scale of *ca.* 48 min while decaying on a time scale of *ca.* 29 min in the 20% glycerol (v/v) solution (Table S1†). The faster decay rate observed in the 20% glycerol (v/v) solution should lead to an increased photoproduct yield and thus contradicts the increased Fe(ii) CN stretch absorbance observed in the IR results in Tris buffer (Fig. 2c) which themselves agree with literature observations.^42,51,54^ However, in both cases, the decay of the ferricyanide absorption band is followed by a long-term gradual drift and growth in the absorbance (potentially due to thermally induced spectral shifts). This drift doesn't correspond to any clear spectral shape changes (Fig. S10b and c†) and complicates kinetic analysis thus significantly lowering the accuracy of the lifetimes estimated here. Consequently, more, strictly controlled, experiments are required to accurately quantify the various rate processes and thus any quantum yields. Nonetheless, the weak absorbance of the photoproduct beyond 500 nm represents a complication for spectral monitoring of photogenerated radicals in flavoproteins which are typically detected in this region.

The above results detail how ferricyanide can undergo photoinduced degradation under weak, off-peak, illumination, under a variety of aqueous conditions. Additional irradiation experiments of ferricyanide dissolved in DMSO (ESI Fig. S12†) showed clear degradation in the UV/Vis detected experiments but without photoreduction of the iron centre. The absorption spectrum of the degradation product matches the products formed neither in deionised water nor in glycerol. After extended irradiation in DMSO the UV-Vis spectrum shows weak absorbance below 500 nm with a sharp and strong increase below 300 nm consistent with Fe^3+^_(aq)_. The absence of Fe(ii) complex production in this case indicates direct loss of a cyanide from the ferricyanide. This provides further evidence that that the solvent medium plays a key role in the ferricyanide photodegradation mechanism (*i.e.* CN^−^*vs*. CN˙ loss) in addition to the excitation wavelength characterised previously.^38^

## 5. Conclusions

In summary, we have shown that the photochemistry ferricyanide undergoes under experimental conditions regularly employed in the study of flavoproteins is markedly different to that in pure water. Specifically, the presence of glycerol results in different, more photostable products albeit accompanied by similar reduction of the metal centre and solvent–ligand exchange. The mechanism and identity of the degradation products depends on the solvent system used with a different product formed depending on the available binding molecules present in the solvent and the mechanism of ligand dissociation.

The observed ferricyanide photochemistry represents a complicating factor for spectroscopic studies of flavoproteins.^18,26^ It remains to be determined whether this photochemistry occurs independently to that of the proteins studied in its presence or directly influences them. If photoexcitation of ferricyanide leads to ligand dissociation then it is reasonable to assume that these coordinatively unsaturated reactive intermediates are capable of binding to proteins in solution *via* surface amino acid residues such as cysteine. This, in turn, could influence protein folding and/or induce aggregation, both of which will influence protein measurements. Therefore, we conclude that the photoinduced degradation of added ferricyanide must be considered with care when analysing the results of protein photochemical studies.

## Author contributions

PDFM performed the experiments under the supervision of PRM. PDFM, PRM, CRT and SRM conceived the study and directed the research. PDFM, SRM and PRM wrote the manuscript which all authors have commented on.

## Data availability

Data supporting the findings of this study are available in the article and the associated ESI.† The ESI also contains additional information about the experimental set-ups, kinetic analysis and DFT calculations. Additional references are cited within the ESI.†

## Conflicts of interest

There are no conflicts to declare.

## Supplementary Material

DT-054-D4DT02916J-s001
