# Supplementary material for: Blue-light photodegradation of ferricyanide under protein relevant conditions
Source: Dalton Trans. 2025 Feb 7;54(11):4735–42. doi: 10.1039/d4dt02916j (PMC11837904; doi:10.1039/d4dt02916j)
Supplement: DT-054-D4DT02916J-s001 [file DT-054-D4DT02916J-s001.pdf]

Supplementary Information (SI) for Dalton Transactions.
This journal is © The Royal Society of Chemistry 2025

Blue-light photodegradation of ferricyanide under protein relevant conditions

Patrick D. F. Murton,[a] Christiane R. Timmel,[a] Stuart R. Mackenzie,*[a] and Patricia Rodriguez-Maciá*[b,c]

[a] *Department of Chemistry, University of Oxford, Chemistry Research Laboratory, Mansfield Road, Oxford, OX1 3TA, UK*

[b] *Department of Chemistry, University of Oxford, Inorganic Chemistry Laboratory, South Parks Road, Oxford, OX1 3QR, UK.*

[c] *School of Chemistry and Leicester Institute for Structural and Chemical Biology, University of Leicester, University Road, Leicester, LE1 7RH.*

Supporting Information

A. Broadband Cavity Enhanced Absorption Spectroscopy (BBCEAS)

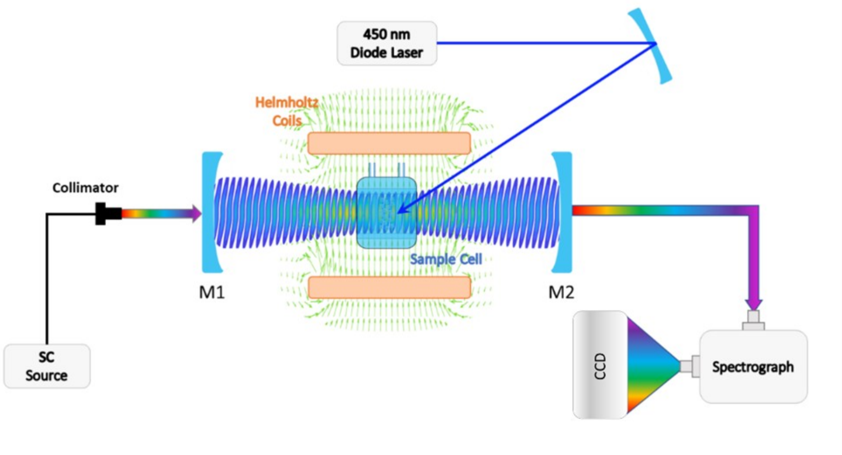

Fig. S1 – Broad-band cavity enhanced absorption spectroscopy experiment schematic. The working principles of this technique have been discussed in detail elsewhere.[1,2] In brief, a sample is placed in an optical cavity (between two opposite facing mirrors). The optical cavity and sample are probed by a white light super-continuum laser (probe) with the light leaving the back cavity mirror (M2) directed into a spectrograph and CCD camera, thus measuring sample absorbance. Simultaneous continuous photoexcitation from a diode laser (pump) induces photoproduct formation, detected as changes in the light intensity exiting M2. The optical cavity forces the probe beam to pass through the sample many times thus enhancing the sensitivity of this technique to small changes in sample absorbance.

B. Reference Spectra

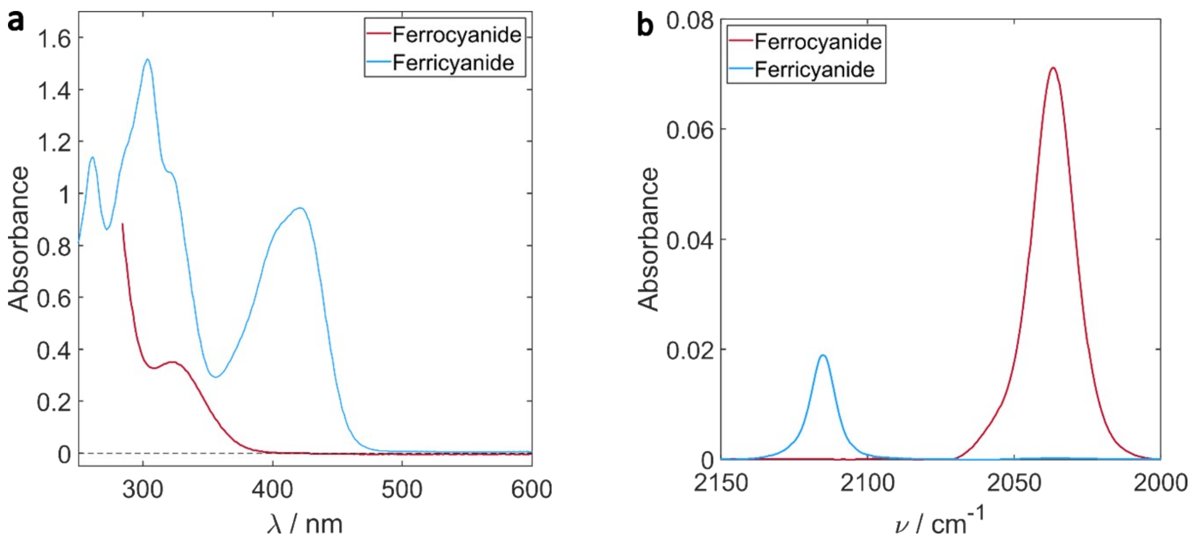

Fig. S2 – Reference spectra for ferricyanide and ferrocyanide. a) UV/Vis spectra of 1 mM ferricyanide, Fe(III), and 1 mM ferrocyanide, Fe(II), in pH 8 Tris buffer (10 mM tris, 250 mM NaCl) with 20 % glycerol (V/V). b) 5 mM ferricyanide and 5 mM ferrocyanide IR spectra in pH 8 tris buffer (10 mM tris, 250 mM NaCl) with 20 % glycerol (V/V). In both cases the IR peaks represent 1T1u CN stretches with the ferricyanide (Fe(III)) peak at 2115 cm^{-1} and ferrocyanide (Fe(II)) peak at 2037 cm^{-1}. The red shift between the two Fe oxidation states arises from the greater π*-back-donation in ferrocyanide leading to a weaker CN bond.

C. BBCEAS spectra of ferricyanide photochemistry

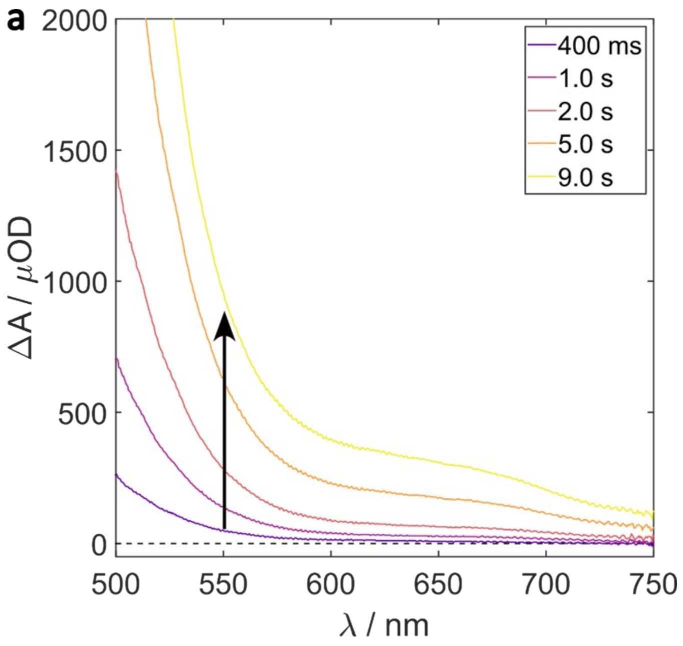

Fig. S3 – BBCEAS ΔA spectra of 5 mM potassium ferricyanide in pH 8 tris buffer (10 mM tris, 250 mM NaCl) with 20 % glycerol (V/V), continuously excited at 450 nm with 3.8 kW m^{-2} irradiance. Clear formation and accumulation of some photoproduct, whose absorbance peaks below 500 nm, over the 10 s illumination period. Back arrow indicates direction of spectral change as a function of time.

D. UV-Vis photoillumination experiments

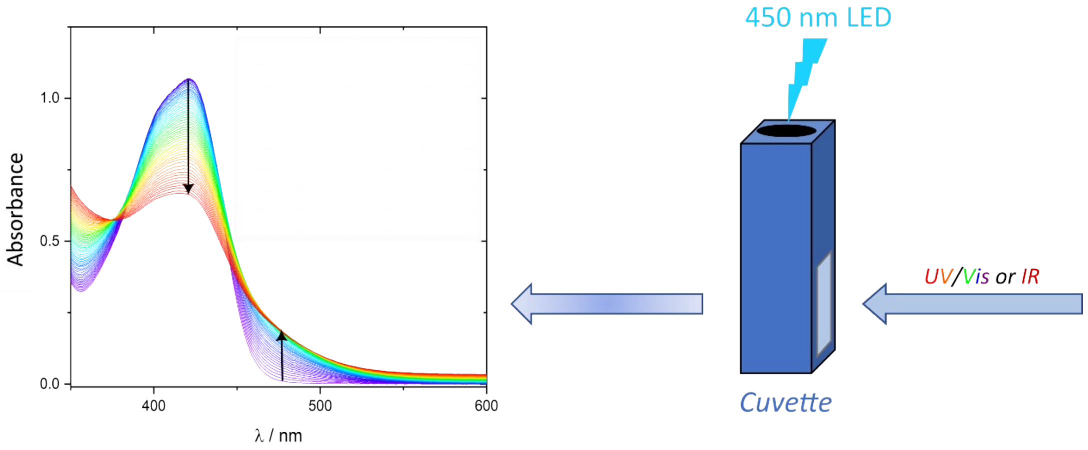

Fig. S4 – Simple schematic of the experimental set-up used in the irradiation experiments. A sample is continuously illuminated *via* a 450 nm LED while a spectrometer periodically probes the sample, measuring its absorbance, in the UV-Vis or IR regions of the spectrum. The data is then passed to a computer and analysed in Matlab. The spectra on the left of the schematic are from a 1 hr illumination of ferricyanide in Tris buffer with a spectrum taken every minute.

E. Ferricyanide in the dark

I. UV-VIS

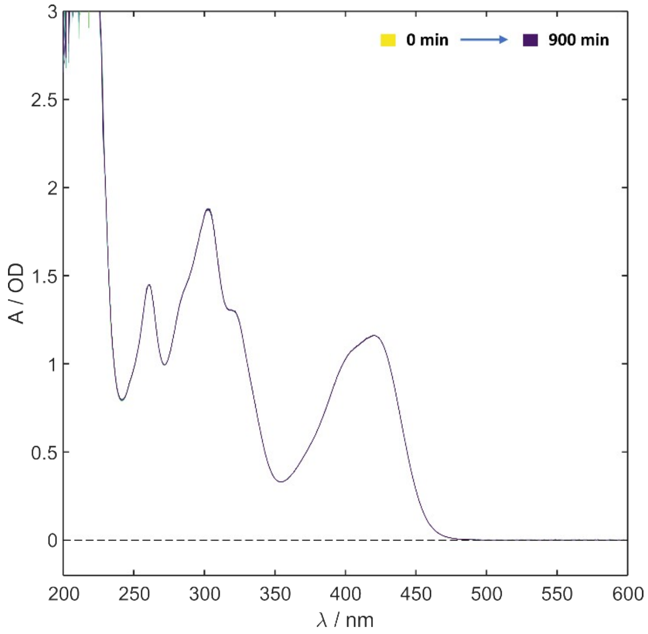

Fig. S5 – UV/Vis spectra of 1 mM potassium ferricyanide in deionised water over a period of 15 hr in the absence of 450 nm photoexcitation. The sample was prepared and maintained in the dark throughout the experiment (with the exception of the probe beam). A spectrum was taken every 30 min with no measurable degradation observed within the measurement time. Potassium ferricyanide is thus highly stable in deionised water in the absence of illumination.

II. Infrared

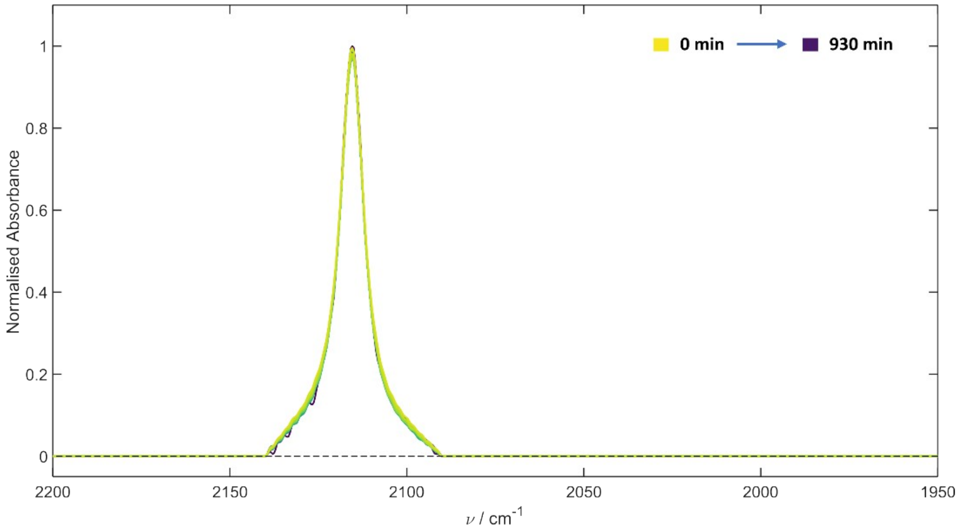

Fig. S6 - IR spectra of 5 mM potassium ferricyanide in deionised water over a period of 15.5 hr in the absence of 450 nm photoexcitation. The sample was prepared in the dark and covered in aluminium foil throughout the experiment with all lab lights turned off. A spectrum was taken every 15 min with no clear degradation observed within the measurement time, confirming potassium ferricyanide's high stability in deionised water in the absence of illumination

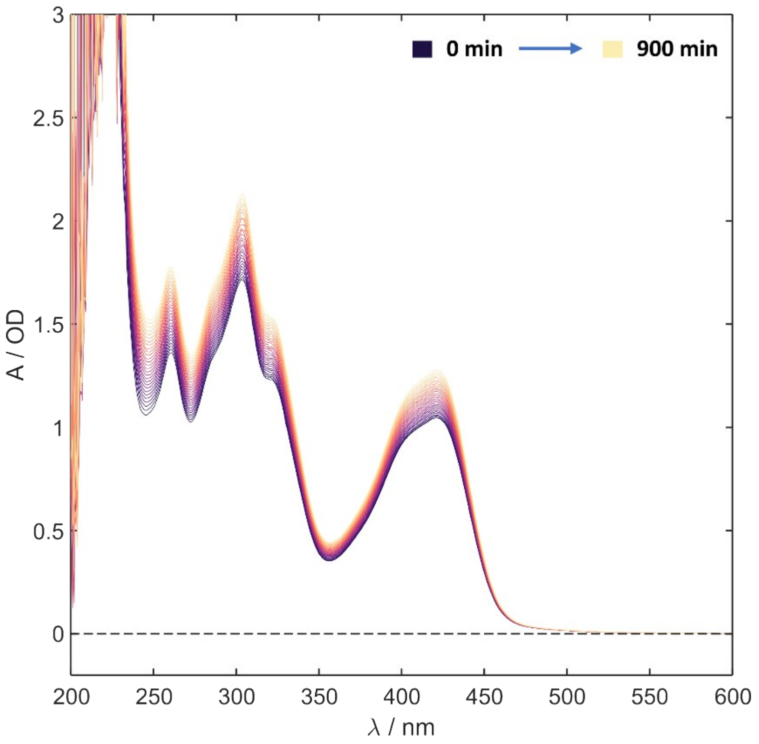

Fig. S7 – UV/Vis spectra of 1 mM potassium ferricyanide in pH 8 tris buffer (10 mM tris, 250 mM NaCl) with 20 % glycerol (V/V). Minimal spectral evolution is observed over a period of 15 hr, in the absence of 450 nm photoexcitation, with a spectrum taken every 30 min. Importantly, the minor spectral evolution observed differs in behaviour to that under 450 nm illumination in the main text. The sample was kept, and prepared, in the dark throughout the experiment.

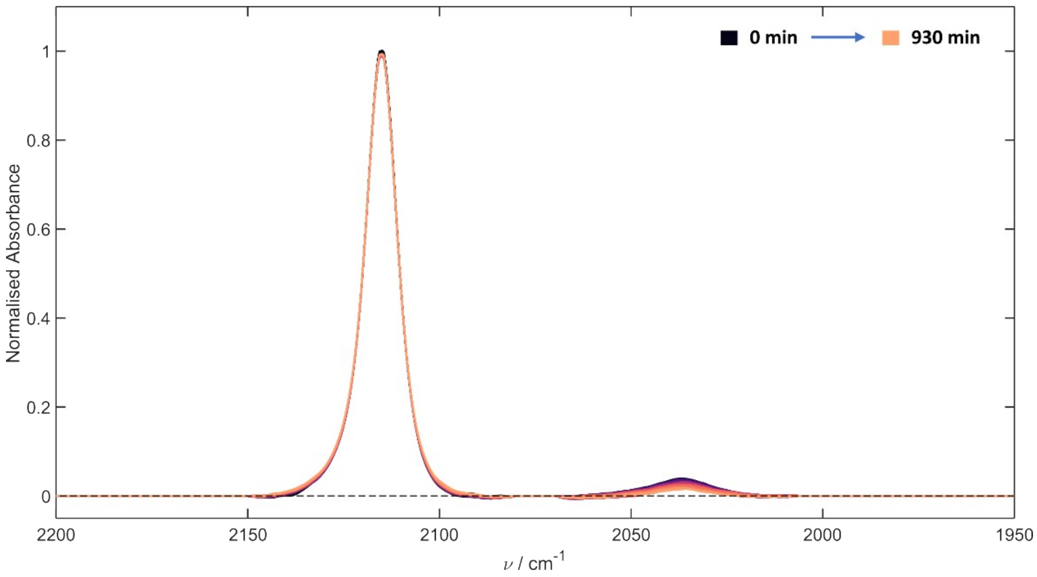

Fig. S8 – IR spectra of 5 mM potassium ferricyanide in pH 8 tris buffer (10 mM tris, 250 mM NaCl) with 20 % glycerol (V/V). Minimal spectral evolution is observed over a period of 15 hr, in the absence of 450 nm photoexcitation, with a spectrum taken every 15 min. A small concentration of Fe(II) is present at t = 0 which appears to decay into Fe(III) in contrast to the behaviour observed for the sample subjected to 450 nm illumination in the main text. The sample was prepared in the dark and covered in aluminium foil throughout the experiment with all lab lights turned off.

F. Tris and glycerol structures

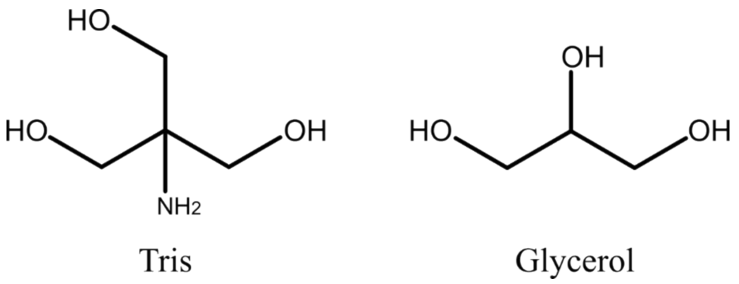

Fig. S9 – Chemical structures of tris(hydroxymethyl) (tris) and glycerol where each can in principle ligate *via* any of their heteroatoms.

G. UV/Vis irradiation kinetics at 420 nm

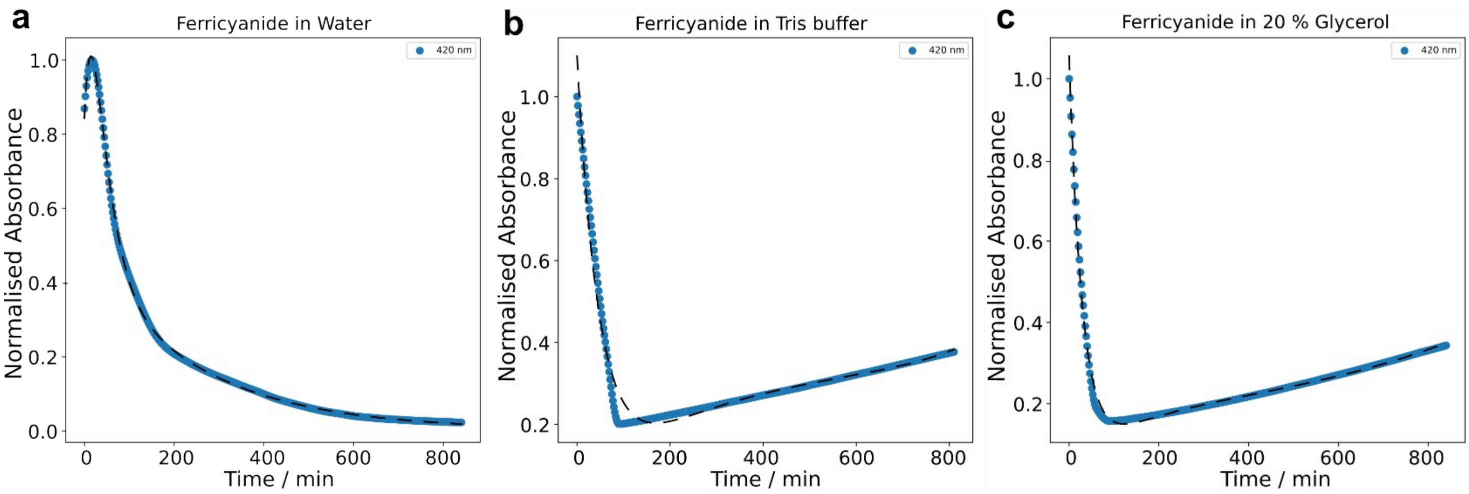

Fig. S10 – Kinetic evolution of the UV/Vis irradiation results at 420 nm, interpolated using SciPy's shape preserving pchip interpolator. a) Normalised absorbance of ferricyanide in MiliQ water *vs.* time. b) Normalised absorbance of ferricyanide in Tris buffer *vs.* time. c) Normalised absorbance of ferricyanide in 20 % glycerol *vs.* time. The black dashed lines in all traces indicate fits to a tri-exponential function (defined below).

All kinetic evolutions displayed in **Fig. S10** were interpolated using a shape preserving pchip interpolator from the python SciPy library before least-square fitting to a tri-exponential function using SciPy's curve fit function. The UV/Vis irradiation kinetics of potassium ferricyanide in MiliQ water, at 420 nm, was fitted to

$$y(t \,|\, A_1,\tau_1,A_2,\tau_2,A_3,\tau_3,C) = A_1 e^{\left(-t/\tau_1\right)} + A_2 e^{\left(-t/\tau_2\right)} + A_3 e^{\left(-t/\tau_3\right)} + C, \tag{S1}$$

while the kinetics of potassium ferricyanide in Tris buffer and 20 % glycerol were both fitted to

(S2)

$$y(t \mid A_1,\tau_1,A_2,\tau_2,A_3,\tau_3) = A_1 e^{\left(-t/\tau_1\right)} + A_2 e^{\left(-t/\tau_2\right)} + A_3\left(1 - e^{\left(-t/\tau_3\right)}\right).$$

As discussed in the main text, the kinetic evolution of ferricyanide in water, at 420 nm (**Fig. S10 a**), exhibits an initial rise in absorbance as ferricyanide is converted into an intermediate, possibly $[Fe(II)(CN)_5(H_2O)]^{3-}$, before subsequently decaying by continued photolysis. This behaviour is successfully captured by equation S1 with the intermediate species formed with a lifetime between 20 – 30 minutes before slowly decaying with a lifetime of *ca.* 245 minutes. In contrast, the kinetic evolution of ferricyanide in Tris buffer and 20 % glycerol largely consists of the simple decay in ferricyanide. However, in both cases there is a long-term gradual drift and growth in the absorbance which doesn't correspond to any significant spectral changes (*c.f.* **Fig. 2** and **3 a**). Since the temperature of the sample was not controlled during irradiation, this drift could arise from sample/solvent heating induced spectral drifts. Unfortunately, this complicated the kinetic fitting of these experiments, especially the sample in Tris buffer, and thus more stringently controlled experiments are required to accurately quantify and characterise the irradiation dynamics under these conditions. Nonetheless, the first term in equation S2 largely captures the decay of potassium ferricyanide while the second two terms combine to capture the slow gradual drift and growth in the spectra and likely have little physical significance. The fit results for all three samples are summarised in **Table S1** below.

Fit Parameters	Water	Tris buffer	20 % glycerol
A_1	0.473 ± 0.009	1.098 ± 0.008	1.020 ± 0.009
τ_1 / min	245.211 ± 6.085	48.355 ± 0.787	29.385 ± 0.406
A_2	3.351 ± 1.842	0.000 ± 0.001	0.035 ± 0.009
τ_2 / min	29.067 ± 2.924	-153.560 ± 72.856	-478.595 ± 51.370
A_3	-2.983 ± 1.848	0.332 ± 0.022	0.147 ± 0.014
τ_3 / min	20.557 ± 2.236	236.512 ± 24.236	131.842 ± 5.113
C	0.003 ± 0.002	n.a.	n.a.

Table S1 – Results from tri-exponential fitting to the UV/Vis kinetic evolution results shown in **Fig. S10**. Fit functions defined by equations S1 and S2. Errors determined from the square root of the diagonal components of the covariance matrix.

H. Simulated IR spectra of ferricyanide, ferrocyanide and plausible degradation products

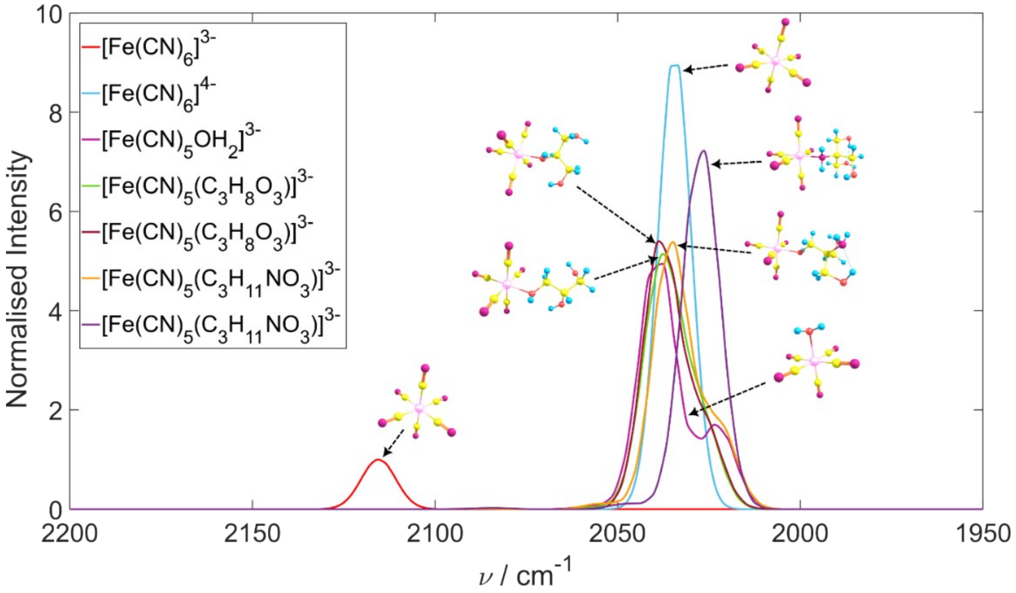

Fig. S11 – DFT simulated IR spectra for potassium ferrocyanide, potassium ferricyanide and potential ferricyanide photodegradation products following continuous 450 nm illumination. All IR peaks arise due to CN stretches with some species exhibiting shoulders or split peaks due to structural shifts reducing symmetry and thus CN stretch degeneracy. All peaks have been shifted by the same factor (1.037) such that the ferricyanide stretch sits at the experimental CN stretch frequency of 2115 cm^{-1}. All of the Fe(II) species exhibit stretches in the region around 2037 cm^{-1}, in agreement with the experimental irradiation results. This highlights that it may not be possible to discern these species apart from their steady-state IR stretches, experimentally.

I. Calculated (DFT) ligand binding energies

Bonds	Binding Energy (eV)
Fe(III) – CN^-	-1.945264472
Fe(II) – CN^-	-1.402723724
Fe(II) – Water	-0.640979109
Fe(II) - Glycerol (end)	-0.577024434
Fe(II) - Glycerol (mid)	-0.542835838
Fe(II) - Tris (Oxygen)	-0.751809802
Fe(II) - Tris (Nitrogen)	-0.980624095

Table S2 – DFT calculated ligand binding energies. Binding energies were calculated as the total energy of the full molecule minus the total energy of the dissociating ligand and penta-coordinated intermediate. All molecules, intermediates and ligands were calculated in aqueous (SMD) solution. Ionic strength and glycerol content was not considered here and may change the ordering of relative bond energies. For the glycerol bond energies, (end) refers to binding *via* a terminal hydroxy oxygen while (mid) refers to binding *via* the secondary hydroxy oxygen. Similarly, for the Tris binding energies,

(Nitrogen), refers to binding *via* the amine nitrogen while, (Oxygen), refers to binding *via* a hydroxy oxygen.

J. Ferricyanide photochemistry in DMSO

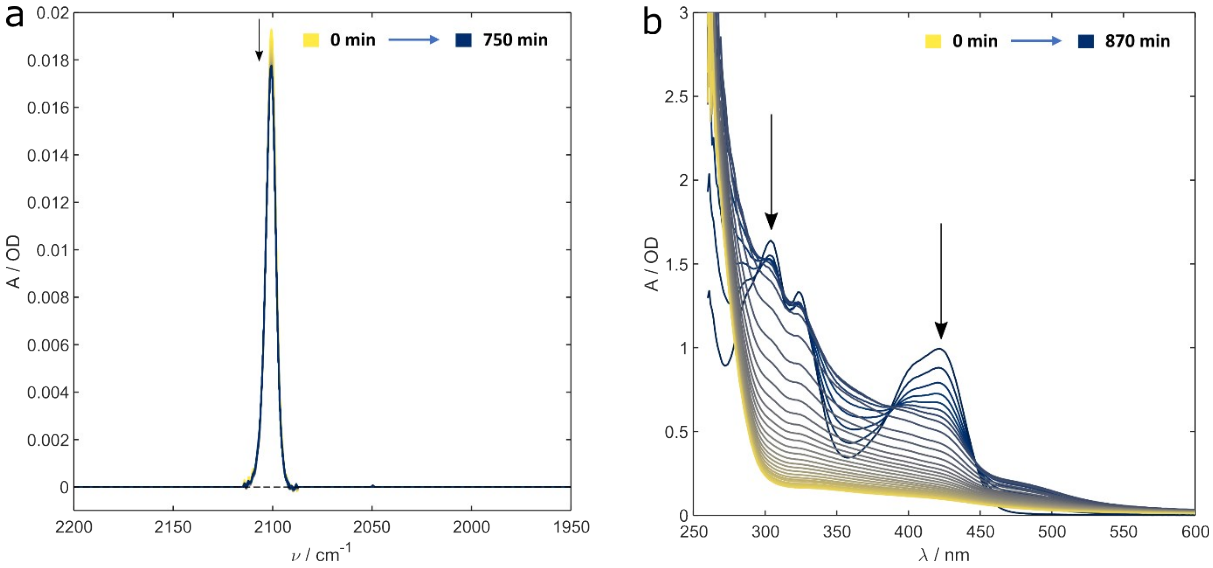

Fig. S12 – Ferricyanide in DMSO irradiation results. a) IR detected irradiation exhibiting a single Fe(III) CN stretch band centred at 2101 cm^{-1} which decays slightly during illumination. No Fe(II) formation was observed. b) UV/Vis detected irradiation shows clear degradation of ferricyanide due to photoexcitation. Thus degradation occurs even in the absence of Fe(II) formation, potentially *via* CN^- loss.

References:

1 S. R. T. Neil, J. Li, D. M. W. Sheppard, J. Storey, K. Maeda, K. B. Henbest, P. J. Hore, C. R. Timmel and S. R. Mackenzie, *J. Phys. Chem. B*, 2014, **118**, 4177–4184.

2 J. Xu, L. E. Jarocha, T. Zollitsch, M. Konowalczyk, K. B. Henbest, S. Richert, M. J. Golesworthy, J. Schmidt, V. Déjean, D. J. C. Sowood, M. Bassetto, J. Luo, J. R. Walton, J. Fleming, Y. Wei, T. L. Pitcher, G. Moise, M. Herrmann, H. Yin, H. Wu, R. Bartölke, S. J. Käsehagen, S. Horst, G. Dautaj, P. D. F. Murton, A. S. Gehrckens, Y. Chelliah, J. S. Takahashi, K.-W. Koch, S. Weber, I. A. Solov'yov, C. Xie, S. R. Mackenzie, C. R. Timmel, H. Mouritsen and P. J. Hore, *Nature*, 2021, **594**, 535–540.
